# Human arm joints reconstruction algorithm in rehabilitation therapies assisted by end-effector robotic devices

**DOI:** 10.1186/s12984-018-0348-0

**Published:** 2018-02-20

**Authors:** Arturo Bertomeu-Motos, Andrea Blanco, Francisco J. Badesa, Juan A. Barios, Loredana Zollo, Nicolas Garcia-Aracil

**Affiliations:** 1 Miguel Hernández University of Elche, Av. Universidad w/n, Ed. Innova, Elche, 03202 Spain; 2 Universidad de Cadiz, Avenida de la Universidad n 10, Puerto Real, 11519 Spain; 3 Research Unit of Biomedical Robotics and Biomicrosystems, Università Campus Bio-Medico di Roma, via Álvaro del Portillo, 21, Roma, 00128 Italy

**Keywords:** Neuro-rehabilitation therapy, End-effector robots, Kinematic reconstruction, Upper limbs

## Abstract

**Background:**

End-effector robots are commonly used in robot-assisted neuro-rehabilitation therapies for upper limbs where the patient’s hand can be easily attached to a splint. Nevertheless, they are not able to estimate and control the kinematic configuration of the upper limb during the therapy. However, the Range of Motion (ROM) together with the clinical assessment scales offers a comprehensive assessment to the therapist. Our aim is to present a robust and stable kinematic reconstruction algorithm to accurately measure the upper limb joints using only an accelerometer placed onto the upper arm.

**Methods:**

The proposed algorithm is based on the inverse of the augmented Jaciobian as the algorithm (Papaleo, et al., Med Biol Eng Comput 53(9):815–28, 2015). However, the estimation of the elbow joint location is performed through the computation of the rotation measured by the accelerometer during the arm movement, making the algorithm more robust against shoulder movements. Furthermore, we present a method to compute the initial configuration of the upper limb necessary to start the integration method, a protocol to manually measure the upper arm and forearm lengths, and a shoulder position estimation. An optoelectronic system was used to test the accuracy of the proposed algorithm whilst healthy subjects were performing upper limb movements holding the end effector of the seven Degrees of Freedom (DoF) robot. In addition, the previous and the proposed algorithms were studied during a neuro-rehabilitation therapy assisted by the ‘PUPArm’ planar robot with three post-stroke patients.

**Results:**

The proposed algorithm reports a Root Mean Square Error (RMSE) of 2.13*cm* in the elbow joint location and 1.89*cm* in the wrist joint location with high correlation. These errors lead to a RMSE about 3.5 *degrees* (mean of the seven joints) with high correlation in all the joints with respect to the real upper limb acquired through the optoelectronic system. Then, the estimation of the upper limb joints through both algorithms reveal an instability on the previous when shoulder movement appear due to the inevitable trunk compensation in post-stroke patients.

**Conclusions:**

The proposed algorithm is able to accurately estimate the human upper limb joints during a neuro-rehabilitation therapy assisted by end-effector robots. In addition, the implemented protocol can be followed in a clinical environment without optoelectronic systems using only one accelerometer attached in the upper arm. Thus, the ROM can be perfectly determined and could become an objective assessment parameter for a comprehensive assessment.

**Electronic supplementary material:**

The online version of this article (10.1186/s12984-018-0348-0) contains supplementary material, which is available to authorized users.

## Background

Robot-assisted therapies have become a new tool in post-stroke upper limb treatments [1, 2]. One of the most common consequences of stroke, brain cells damage caused by an interruption of the blood flow to the brain, is the hemiparesis, a loss of physical strength on one side of the body, as well as memory problems that they directly affect the realization of the Activities of Daily Living (ADL) [3]. The main goal in these kind of therapies is the effective use of neuroplasticity of the brain performing several exercises assisted by a robotic device which can be adapted to the tasks regarding his/her residual motor capabilities. This technology aims to maximize the patient’s recovery, minimize the rehabilitation period and encourage the motivation of patients [4–6].

**Fig. 1:**
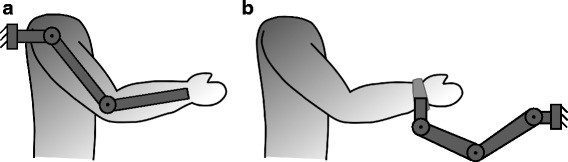
Robotic devices for upper limb rehabilitation: **a** Exoskeletons, **b** End effector

Rehabilitation robotic devices for upper limbs can be classified into two types: exoskeletons devices [7], have robot axes aligned with the anatomical axes of the upper limb segments providing direct control of individual joints, and end-effector devices [8], work by applying mechanical forces to the distal segments of limbs (see Fig. 1). Though exoskeletons allow the total control of the arm joints, they are difficult to adapt and attach to the patient arm [9, 10]. Moreover, the attachment process takes a long time in order to avoid misalignment between the robot and the arm that can injure the patient. However, end-effector robots can be easily adapted and used by several patients with different pathologies [11–14]. Nevertheless, these robots provide information about the end effector trajectory followed during the therapy and the interaction forces between the hand and the end effector, by which the therapist can perform an objective assessment and customize the therapy based on patients’ needs [15–17], but they are not able to know the upper limb joints of the patient.

On the other hand, monitoring joint angles enables human posture to be analyzed in a wide range of application and disciplines, such as physical and neuro-rehabilitation, sports medicine or virtual training. The Range of Motion (ROM) in upper limb neuro-rehabilitation therapy offers a comprehensive assessment together with the clinical assessment scales [18–20]. Standard motion analysis instruments are widely used in these fields that can be mainly divided into three groups: optoelectronic systems, inertial measurement units (IMUs) systems, and wearable goniometers. The former system is often very expensive and difficult to adapt into a clinic environment, it requires a large and controlled area without camera obstruction [21]. The latter is an emerging technology that aims to measure the angle joints by the deformation of a specific sensor or by optical-based goniometers [22–24]. However, they are able to measure only simple joints as a flexo-extension of the knee or the elbow, not a combination of upper limb joints. The IMUs systems, based on the integration of accelerometers, gyroscopes and magnetometers, have gained the reputation of being the cutting edge of wearable motion tracking systems [25, 26]. IMUs estimate the orientation of the body segments where they are attached by combining multi-sensor information through dedicated optimal sensor fusion algorithms. However, the calibration of these sensors is sometimes very difficult to achieve with post-stroke patient due to a specific body configuration requirements, as with the well known XSens MVN system [27], or the system need a fusion of many sensors placed onto the body [28].

There are several studies which have produced arm reconstruction through motion tracking cameras to estimate the position of the arm and implement a visual feedback on rehabilitation activities [29, 30]. However, they do not perform an accurate measurement of the arm joints during the rehabilitation therapy. A new tool capable to compute the arm joints through two non-invasive accelerometers placed onto the upper arm was introduced by Mihelj [31]. Papaleo et al. improved this method by integrating the joint kinematic reconstruction through the inverse of the augmented Jacobian being able to accurately estimate the human upper limb joints using only one accelerometer [32]. Although this algorithm presents a low error with respect to the real arm, it is unstable when a small shoulder movement is done due to the inevitable trunk compensation performed by patients. Furthermore, the system uses the information of an optoelectronic system to measure the upper arm and forearm lengths, the shoulder position, and the initial position.

In this paper, an upper limb kinematic reconstruction algorithm, based on the same criterion presented in [32], is developed. It uses the information provided by one accelerometer placed onto the upper arm and by the end effector of the robot. This algorithm solves the instability in the upper limb joints estimation, proposing a protocol to manually measure the upper arm and forearm lengths and we present a technique to estimate the initial upper limb joints. The main difference between the proposed and the previous algorithm is that the estimation of the elbow joint location is done through computation of the accelerometer rotation after an arm displacement. The end-effector robot with seven Degrees of Freedom (DoF), designed and built by the Neuro-Bioengineering Research Group (nBio), Miguel Hernández University of Elche, Spain, was used to carry out the experimental validation of the proposed algorithm [33]. Furthermore, a comparative analysis of both algorithms in a neuro-rehabilitation therapy with post-stroke patients is performed, studying their behavior when shoulder movements cannot be avoided by patients but measured through the method proposed in [34] using the ‘PUPArm’ robot.

## Methods

### Kinematic model of the human arm

**Fig. 2:**
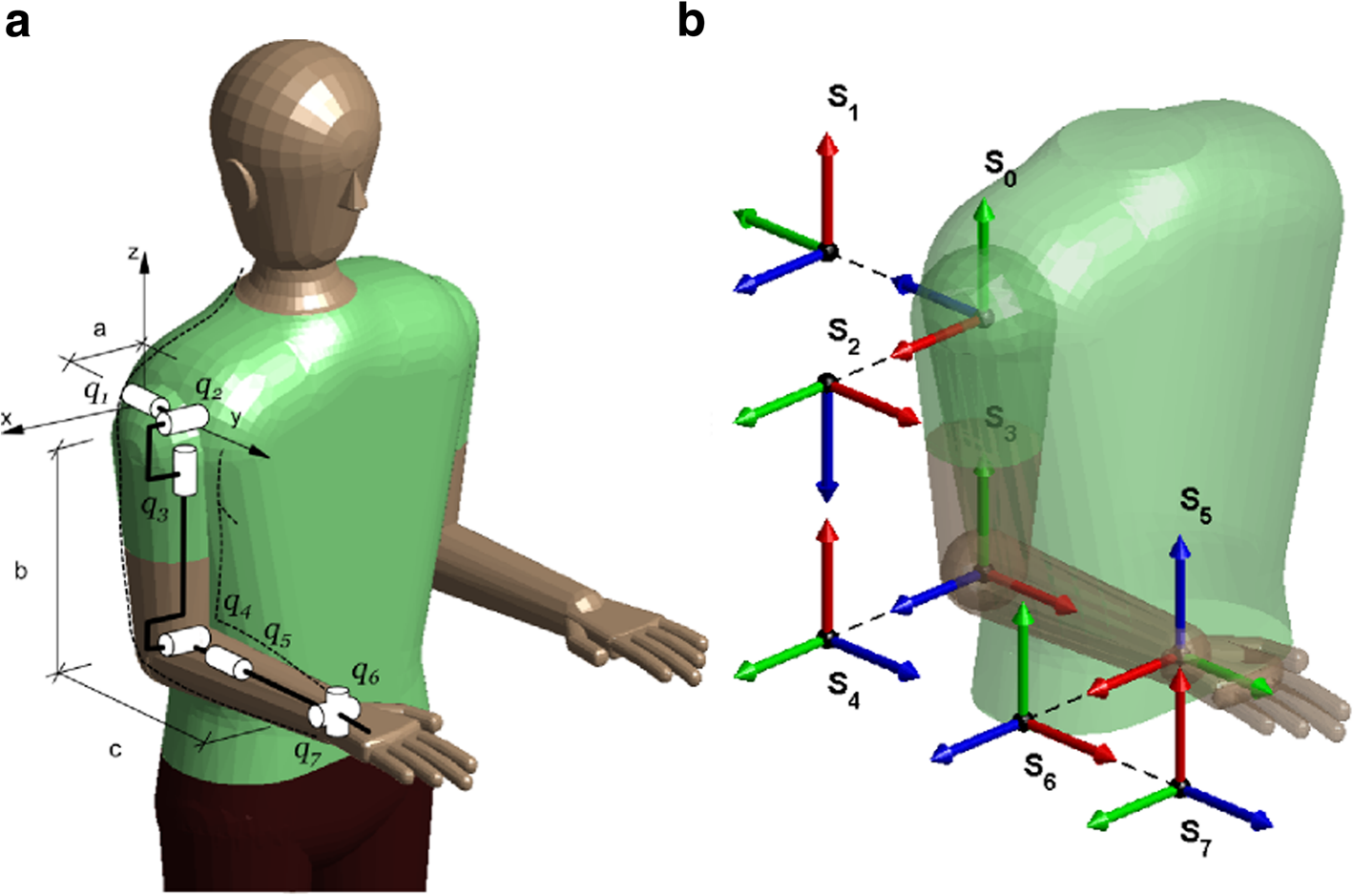
Simplified human arm model. **a** Rotational joints. **b** DH reference systems where *X*, *Y* and *Z* axes are represented by the red, green and blue colors, respectively

The human arm is a complex kinematic chain that can be simplified into seven DoF arm model, connected through two links: upper arm (*l*_*u*_) and forearm (*l*_*f*_), as can be seen in Fig. 2a) [35]. The shoulder has been modeled as a spherical joint composed of abduction-adduction (*q*_1_), flexion-extension (*q*_2_) and internal-external rotation (*q*_3_) movements. The double-hinge elbow joint comprises the flexion-extension (*q*_4_) and pronation-supination (*q*_5_) of the forearm. Though *q*_5_ anatomically belongs to the elbow joint, it is considered as a wrist DoF. Thereby, the wrist joint is a spherical joint composed of *q*_5_, ulnar-radial deviation (*q*_6_) and flexion-extension (*q*_7_) of the hand. The Denavit-Hartenberg (DH) parameters [36] of the arm and the reference systems of each joint were established as are shown in Table 1 and in Fig. 2b, respectively.

**Fig. 3:**
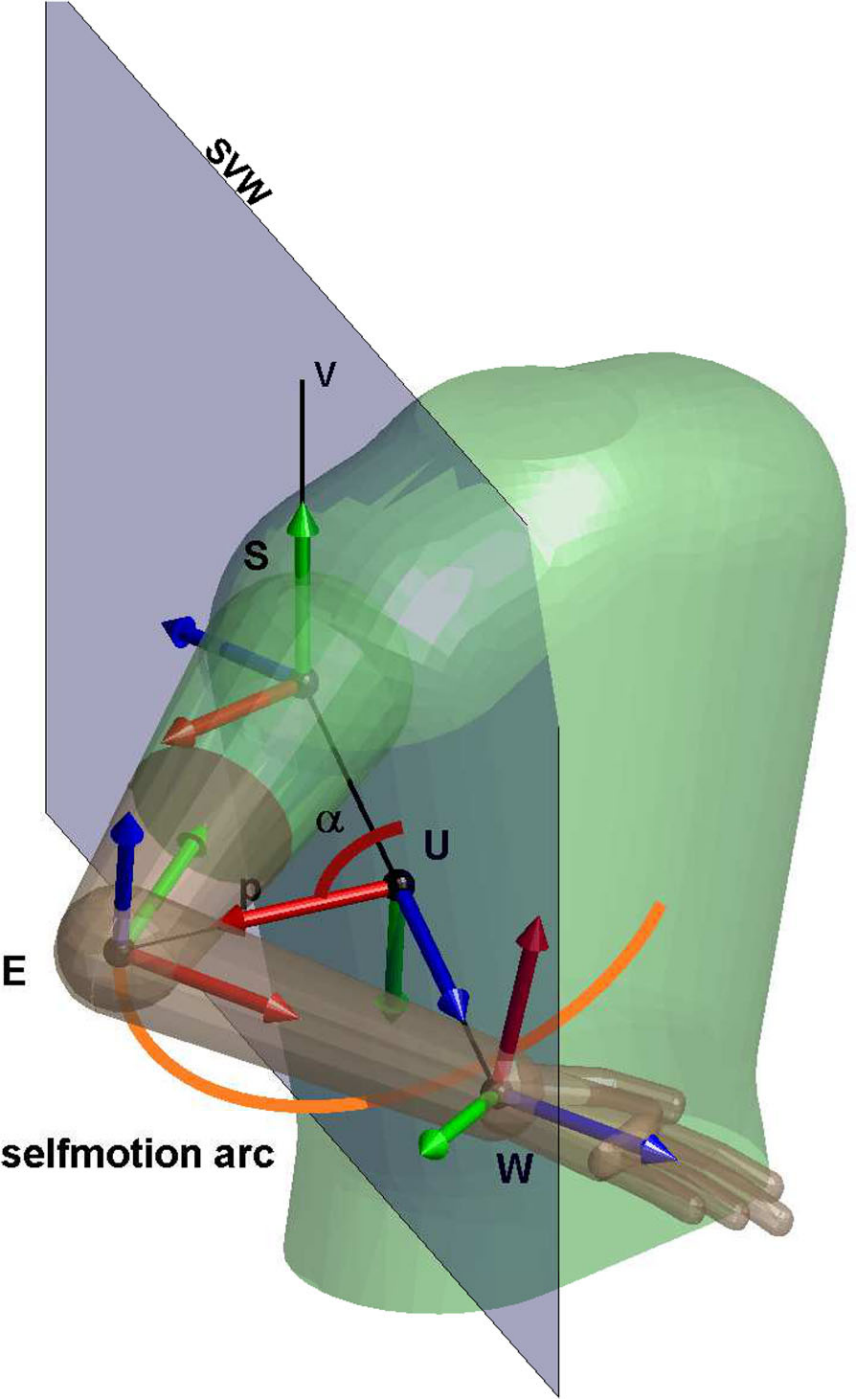
Self motion arc and angle *α* represented on a random position of the arm

**Table 1:** DH Parameters of the human arm

***i***	***θ***_***i***_	***d***_***i***_	***a***_***i***_	***α***_***i***_
1	*π*/2+*q*_1_	0	0	*π*/2
2	3*π*/2+*q*_2_	0	0	*π*/2
3	*q*_3_	*l*_*u*_	0	−*π*/2
4	*π*/2+*q*_4_	0	0	*π*/2
5	*π*/2+*q*_5_	*l*_*f*_	0	*π*/2
6	*π*/2+*q*_6_	0	0	*π*/2
7	*π*/2+*q*_7_	0	0	*π*/2

### Inverse kinematics with augmented Jacobian

The kinematic reconstruction algorithm is based on the augmented Jacobian introduced by Kreutz-Delgado [37]. The analysis of a seven DoF manipulator with revolute joints was performed to uniquely determine the joint angles for a given end-effector location. The redundancy is catheterized by the swivel angle (*α*), the angle between the arm plane formed by the shoulder, elbow and wrist points and a reference plane *SVW*, shown in Fig. 3.

Then, the augmented Jacobian can be expressed as

$$ J_{A}(\vec{q}) = \left[ \begin{array}{c} J_{g}(\vec{q}) \\ J_{\alpha}(\vec{q}) \end{array} \right],  $$

where $J_{g}(\vec {q})$ is the geometric Jacobian matrix of the arm and $J_{\alpha }(\vec {q})$ is the swivel angle Jacobian, providing the joint velocities with respect to the amount of change of *α*. Thus, the arm joint velocities are computed through the inverse of the augmented Jacobian with respect to the upper limb joints ($\vec {q}$) as

1 $$ \dot{\vec{q}} = J^{-1}_{A}\left(\vec{q}\right) \left\lbrace \left[ \begin{array} {c} \dot{\vec{v_{d}}} \\ \dot{\alpha} \end{array} \right] + K \cdot \vec{err}\right\rbrace,   $$

being $\dot {\vec {v_{d}}}$ the hand velocity vector and $\dot {\alpha }$ the swivel angle velocity. The error produced by the discrete integration is minimized with the vector error ($\vec {err}$) multiplied by a suitable gain matrix *K* [38]. The Jacobian matrix can induce high joint speed in the regions close to kinematic chain singularities. Thereby, the damped least-square approach [38] was applied to the augmented Jacobian matrix as

$$ J^{*}_{A} = J_{A}^{T} \left(J_{A}\cdot J^{T}_{A}+k^{2}\cdot I\right)^{-1}, $$

where *k*^2^ is the damping factor that, chosen properly, performs an accuracy approach to the singularity area, and *I* is the identity matrix. Therefore, the Jacobian matrix $J^{*}_{A}$ is introduced in (1) instead of *J*_*A*_.

Thus, the arm joints at time *t*_*k*_ are estimated as

$$ \vec{q}(t_{k})= \vec{q}(t_{k-1})+\dot{\vec{q}}(t_{k})\Delta t,  $$

being $\vec {q}(t_{k-1})$ the previous arm joints, $\dot {\vec {q}}$ computed from (1) and *Δt* the sampling rate.

### Elbow estimation

The estimation of the elbow joint pose is the key of the proposed inverse kinematic reconstruction. It is computed through the orientation of the accelerometer placed onto the upper arm. This orientation can be estimated assuming slow movements during the exercise, to erase the dynamic component of the acceleration.

**Fig. 4:**
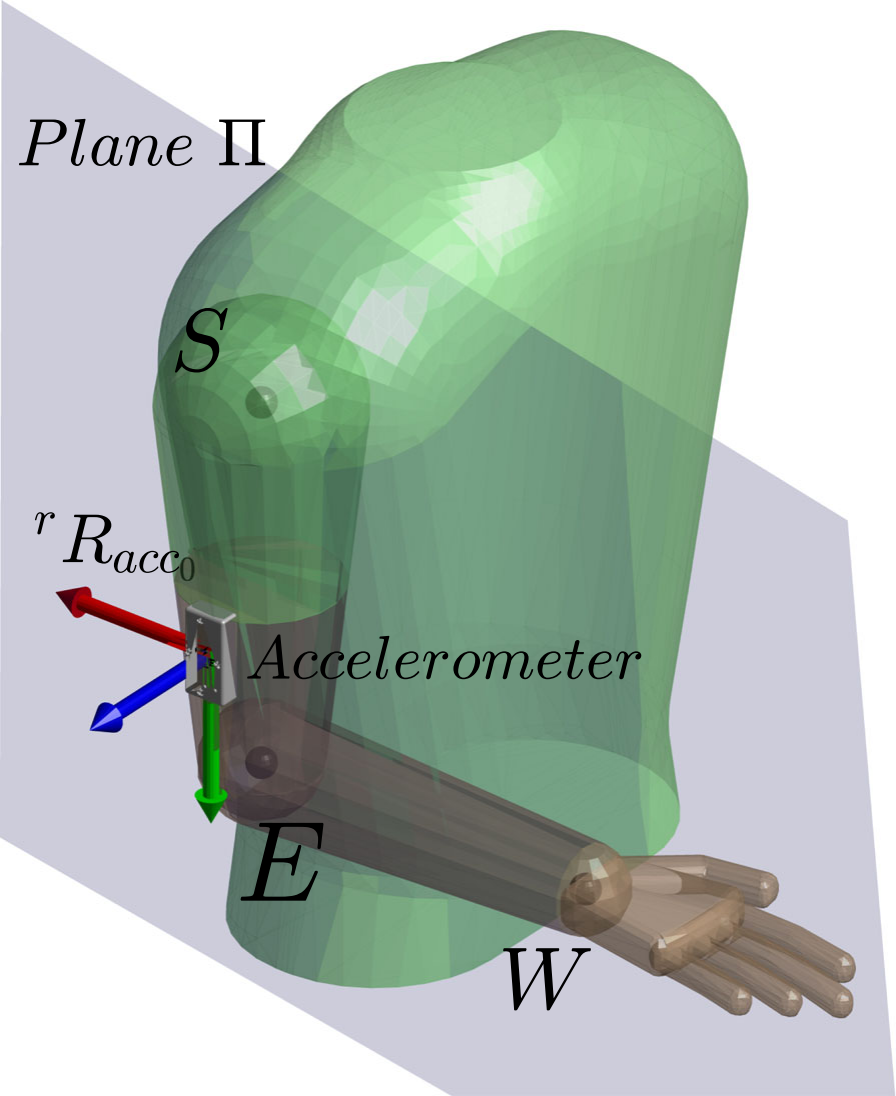
Reference position of the arm and reference orientation of the accelerometer where *X*, *Y* and *Z* axes are represented by the red, green and blue colors, respectively

Starting from the reference position of the arm and the accelerometer, shown in Fig. 4, the value of the accelerometer at this position, normalized with respect to the gravity acceleration, is

$$ {{~}^{acc_{0}}}V_{g} = \left[ \begin{array}{c} 0 \\ 1 \\ 0 \\ \end{array}\right].  $$

The acceleration value in a random upper limb position can be expressed as a combination of the reference value and the rotation applied ($\phantom {\dot {i}\!}{{~}^{acc_{0}}}R_{acc}$) as

$$ {{~}^{acc}}V_{g} = \left({{~}^{acc_{0}}}R_{acc}\right)^{-1} {{~}^{acc_{0}}}V_{g}.  $$

The rotation matrix $\phantom {\dot {i}\!}{{~}^{acc_{0}}}R_{acc}$ is unknown, however one possible solution might be computed as

$${{~}^{acc_{0}}}\tilde{R}_{acc} = I + M + M^{2} \frac{1-\cos (\theta)}{\sin^{2} (\theta)},  $$

with

$$ \begin{aligned} M &= \left[ \begin{array}{rrr} 0 & -V(3) & V(2)\\ V(3) & 0 & -V(1)\\ -V(2) & V(1) & 0 \end{array}\right],\\ V &= {{~}^{acc_{0}}}V_{g} \times {{~}^{acc}}V_{g},\\ sin(\theta) &= \|V\|,\\ cos(\theta) &= {{~}^{acc_{0}}}V_{g} \cdot {{~}^{acc}}V_{g}.\\ \end{aligned}  $$

From this rotation, it is possible to find the proper arm position making the plane *XY* of ${{~}^{acc_{0}}}\tilde {R}_{acc}$ to include the known shoulder and wrist joints position, shown as *Π* plane in Fig. 4. Hence, it is necessary to rotate the matrix ${{~}^{acc_{0}}}\tilde {R}_{acc}$ around the gravity vector a *γ* angle to accomplish this restriction. The computation of this angle is explained in the Additional file 1. The simplification performed in order to obtain this angle allows the algorithm to be performed in real time (average time in the computation of the mathematical operations: ≈0.9 *ms* running on the Intel Core i7 3.40GHz with Matlab R2017a).

Two solutions of angle *γ* are found, each solution computes a different rotation matrix ${{~}^{acc_{0}}}R^{(i)}_{acc}$, with *i*∈{1,2}, in which the *Z* axis point to each normal vector of the plane *Π*. Thus, two elbow positions with respect to the robot (^*r*^*P*_*e*_) are obtained as

$$\begin{array}{*{20}l} ^{r}P_{e} &=^{r}T_{acc} \cdot \left[ \begin{array} {cccc} 0&lu&0&1\end{array} \right]^{T} \text{, with}\\  {^{r}T_{acc}} &= \left[ \begin{array}{cc} ^{r}R_{acc_{0}} \cdot {{~}^{acc_{0}}}R^{(i)}_{acc} & {^{r}P_{s}}\\ 0&1 \end{array}\right], \end{array} $$

being ^*r*^*T*_*acc*_ the homogeneous matrix of the accelerometer regarding the robot, $^{r}R_{acc_{0}}$ the rotation matrix between the robot and the accelerometer in the reference position of the arm and ^*r*^*P*_*s*_ the shoulder joint position regarding the robot. Therefore, the correct elbow position is the one which the *Z* axis of the ${{~}^{acc_{0}}}R^{(i)}_{acc}$ points the same direction as the cross product between the segment $\overline {EW}$ and $\overline {ES}$ being *S*, *E* and *W* the shoulder, elbow and wrist joint position.

Finally, the elbow location regarding the robot is estimated as

2 $$\begin{array}{*{20}l} {^{r}T_{e}} &= \left[ \begin{array}{cc} {^{r}}R_{e} & {^{r}P_{e}}\\ 0&1 \end{array}\right] \text{, with}\\  ^{r}R_{e} &=^{r}R_{acc_{0}} \cdot^{acc_{0}}R_{acc} \cdot^{acc_{0}}R_{e}  \end{array} $$

being $\phantom {\dot {i}\!}{~}^{acc_{0}}R_{e}$ the rotation matrix of the elbow regarding the accelerometer in the reference arm position. Once the location of the elbow joint is estimated, the swivel angle, necessary to compute the augmented Jacobian, can be computed [37].

### Initial conditions

The initial upper limb joints are necessary to the kinematic reconstruction algorithm. The following locations with respect to the robot are initially known: the shoulder ^*r*^*T*_*s*_, obtained at the beginning of the therapy; the wrist ^*r*^*T*_*w*_, known through the end effector of the robot; and the elbow ^*r*^*T*_*e*_, estimated as explained in the previous section. Thus, the initial joint angles can be estimated using the DH parameters [39] shown in Table 1.

The known matrix that determines the shoulder movement regarding its joints (*q*_1_, *q*_2_, *q*_3_) is defined as

$$\begin{array}{*{20}l} {^{r}T_{s}} &= {^{s_{0}}T_{s_{3}}} = {^{s_{0}}T_{s_{1}}} \cdot {^{s_{1}}T_{s_{2}}} \cdot {^{s_{2}}T_{s_{3}}}\simeq \left[ \begin{array}{cccc} n_{x} & n_{y} &n_{z} & p_{x}\\ o_{x} & o_{y} &o_{z} & p_{y}\\ a_{x} & a_{y} &a_{z} & p_{z}\\ 0 & 0 & 0 & 1\\ \end{array}\right]; \end{array} $$

and two possible solutions of the shoulder joints are obtained as

$$\begin{aligned} {}\text{(i) if }& q_{2} \in \left[0 \quad \pi \right]: & \text{(ii) if }& q_{2} \in \left[0 \quad \pi \right]: \\ q_{1} &= \text{atan2}\left(-n_{y}, o_{y}\right) & q_{1} &= \text{atan2}\left(-n_{y}, o_{y}\right) \\ q_{2} &= \text{atan2}\left(a_{y},\sqrt{n^{2}_{y}+o^{2}_{y}}\right) & q_{2} &= \text{atan2}\left(\!a_{y},\,-\,\sqrt{n^{2}_{y}+o^{2}_{y}}\right) \\ q_{3} &= \text{atan2}\left(a_{z},-a_{x}\right) & q_{3} &= \text{atan2}\left(-a_{z},a_{x}\right) \\ \end{aligned} $$

On the other hand, the flexion-extension of the elbow, joint *q*_4_, affects the distance $\overline {SW}$ and, therefore, it can be unequivocally computed through the law of the cosines as

$$ q_{4} = \arcsin\left(\frac{l^{2}_{u} + l_{f}^{2} - {||W-S||}^{2}}{2 l_{u} l_{f}} \right).  $$

Finally, since the wrist location is given by the robot end-effector pose, its transformation matrix $\phantom {\dot {i}\!}{^{r}}T_{w} = {^{s_{0}}T_{s_{7}}}$ is known. Thus, the wrist joints can be also estimated following the criterion used to solve the shoulder joints as

$$\begin{array}{*{20}l} {{~}^{s_{4}}T_{s_{7}}} &= \left({{~}^{s_{0}}T_{s_{3}}} \cdot {{~}^{s_{3}}T_{s_{4}}} \right)^{-1} \cdot {{~}^{s_{0}}T_{s_{7}}}\simeq \left[ \begin{array}{cccc} n_{x} & n_{y} &n_{z} & p_{x}\\ o_{x} & o_{y} &o_{z} & p_{y}\\ a_{x} & a_{y} &a_{z} & p_{z}\\ 0 & 0 & 0 & 1\\ \end{array}\right]; \end{array} $$

with ${~}^{s_{3}}T_{s_{4}}\phantom {\dot {i}\!}$ the homogeneous matrix of the joint *q*_4_, and two possible solutions can be also obtained as

$$\begin{aligned} \text{(iii) if }& q_{6} \in \left[-\pi/2 \quad \pi/2 \right]: & \text{(iv) if }& q_{6} \in \left[\pi/2 \quad 3\pi/2 \right]: \\ q_{5} &= -\text{atan2}\left(n_{y}, o_{y}\right) & q_{5} &= \pi - \text{atan2}\left(n_{y}, o_{y}\right) \\ q_{6} &= \text{arcsin}\left(a_{y}\right) & q_{6} &= \pi - \text{arcsin}\left(a_{y}\right) \\ q_{7} &= -\text{atan2}\left(a_{x},a_{z}\right) & q_{7} &= \pi - \text{atan2}\left(a_{x},a_{z}\right) \\ \end{aligned} $$

Thereby, four solutions, two due to the shoulder joints and two due to the wrist joints, can satisfy the kinematic constraints. However, only one solution accomplishes the anatomical features of the human upper limb. This statement is provable because the human arm joints vary in [−*π*/2 *π*/2] and each solution belongs either [0 *π*] range or [0 −*π*] range and, therefore, the initial arm joints remain defined. An extensive explanation of the estimation of the initial conditions is presented in Additional file 2.

### Experimental protocol

Two different experiments were performed, in the first experiment was intended to measure the accuracy of the proposed algorithm with respect to an optoelectronic system, taken as a ground truth, and the second was intended to study the behaviour of the algorithm in a rehabilitation therapy and compare its stability with respect to the previous algorithm presented in [32]. Data recordings have been approved by the ethics committee of the Miguel Hernández University of Elche, Spain. All the subjects provided written informed consent.

The first experimental exercise was carried out by seven right-handed healthy subjects performing three trials, their main information is presented in Table 2. The subjects wore a specific jacket with 25 markers attached to it using the baseline upper body marker set [40] in order to measure the ‘ground truth’ joints. Thus, the location of the upper arm, forearm and hand were directly obtained through the optoelectronic system and therefore the arm joints were computed as explained in the previous section. In order to estimate the upper limb joints through the proposed algorithm, a magneto-inertial sensor was tightly attached to the upper arm and the wrist joint location was obtained with the end-effector robot with seven DoF, designed and built by the Neuro-Bioengineering Research Group (nBio), Miguel Hernández University of Elche, Spain [33]. The shoulder joint location was only measured at the beginning of the experimentation through the optoelectronic system as the shoulder and the trunk are fixed during the exercise. The trajectory was previously established in the end-effector robot, a point to point task.

**Table 2:** Main information of the healthy subjects

ID	Age	Forearm length [m]	Upper arm length [m]
1	24	0.25	0.34
2	31	0.21	0.30
3	24	0.26	0.32
4	26	0.26	0.29
5	29	0.26	0.31
6	24	0.23	0.33
7	25	0.26	0.30

**Fig. 5:**
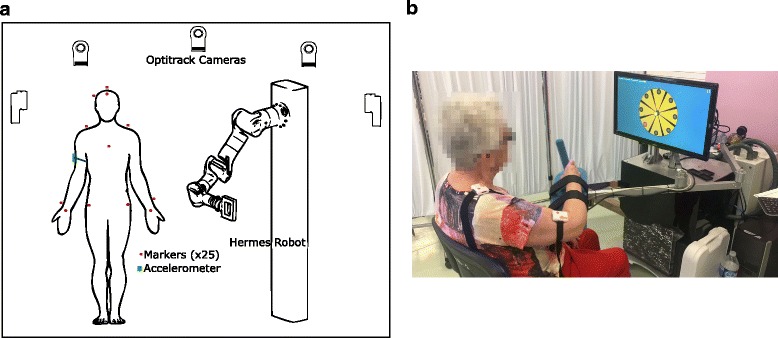
Experimental exercises. **a** Healthy subjects performed an arm movement using a 7 DoF robot wearing an accelerometer placed onto the upper arm and special jacket with optoelectronic markers. **b** Post-stroke patients performed arm movements using the ‘PUPArm’ robot wearing an accelerometer placed onto the upper arm and a magneto-inertial device placed onto the shoulder

The second experimental exercise was carried out by three post-stroke patients, the scores of two assessment scales are shown in Table 3, Ashworth [41], for the elbow joint, and Fugl-Meyer [42]. Two magneto-inertial sensors were used, one attached to the upper arm and the other onto the shoulder (see Fig. 5). The wrist joints location was computed during the exercise with the end-effector robot called ‘PUPArm’, designed and built by the Neuro-Bioengineering Research Group (nBio), Miguel Hernández University of Elche, Spain; and the shoulder joint location, as the flexion-extension and ulnar-radial deviation of the wrist joint is fixed by the robot, the algorithm proposed in [34] can be used and the shoulder location remains estimated during the exercise. The subjects performed three movements in the roulette activity [43].

**Table 3:** Main information of the post-stroke patients

ID	Affected arm	Age	Forearm length [m]	Upper arm length [m]	Fugl-Meyer (A+B+C+D)^*^	Ashworth
1	Right	51	0.24	0.30	(33+7+7+2) /66	2
2	Left	58	0.25	0.33	(27+4+3+3) /66	3
3	Left	74	0.24	0.35	(24+2+2+1) /66	3

^*^Fugl Meyer scale for upper extremity is divided in: A) Upper Extremity, B) Wrist, C) Hand, D) Coordination/speed

In both exercises, the length of the upper arm was manually measured from the lateral side of the acromion to the proximal radius head, in the elbow joint; and the forearm length was measured from the proximal radius head to the radial styloid, the distal part of the radius [44]. Furthermore, the upper limb joints were on-line estimated. The main parameters of the kinematic reconstruction algorithm, the gain matrix and the damping factor, were set to *K*=*diag*{1.5,1.5,…1.5} *N*/*ms* and *k*^2^=0.5 respectively. They were chosen through a “trial and error” approach under the exercise conditions. The magneto-inertial sensors used were developed by Shimmer^™^ and sampled at 100*Hz*. The optoelectronical system was composed by 8 6DoF optical tracking cameras Optitrack V100: R2, developed by NaturalPoint^®;^. This camera has a 640×480 *px* resolution with an approximate precision of 0.3 *mm* and frame rate of 30−120 frames per second.

## Results

### Validation of the proposed algorithm

**Fig. 6:**
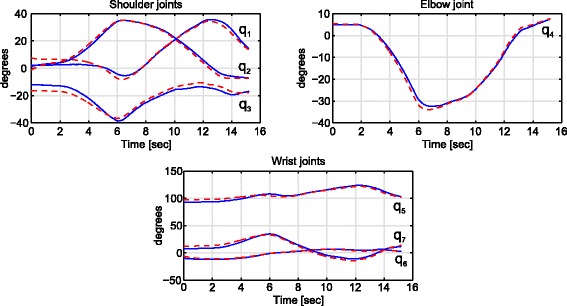
Reconstructed upper limb joints estimated by the proposed algorithm (blue smooth line) and acquired through the optoelectronic system (red dotted line) of one trial performed by a healthy subject

This algorithm was previously studied in a simulated environment with a 7 DoF robot, being able to avoid shoulder movements and misalignment between the accelerometer and the upper arm, in [45]. The accuracy of the proposed algorithm was measured as the difference between the values acquired through the optoelectronic system and estimated by the proposed algorithm in terms of Root Mean Square Error (RMSE), Standard Deviation (SD) and correlation coefficient (R), shown in Table 4. It can be observed that the correlation between both upper limb joints reconstruction is high with low error. In addition, the reconstructed kinematic joints of a subject while performing a trial is shown in Fig. 6.

**Table 4:** RMSE of the proposed algorithm regarding the optoelectronic system reconstruction (grand mean of the seven subjects)

	RMSE	SD	R	*p*-value
Elbow joint position [cm]	2.13	1.1	0.977	< 0.001
Wrist joint position [cm]	1.89	1.12	0.982	< 0.001
*q*_1_ [deg]	3.548	2.148	0.957	< 0.001
*q*_2_ [deg]	3.271	2.992	0.977	< 0.001
*q*_3_ [deg]	4.569	3.041	0.966	< 0.001
*q*_4_ [deg]	1.719	1.542	0.995	< 0.001
*q*_5_ [deg]	4.506	2.741	0.873	< 0.001
*q*_6_ [deg]	2.825	2.226	0.899	< 0.001
*q*_7_ [deg]	4.742	3.279	0.982	< 0.001

### Experimental results with patients

**Fig. 7:**
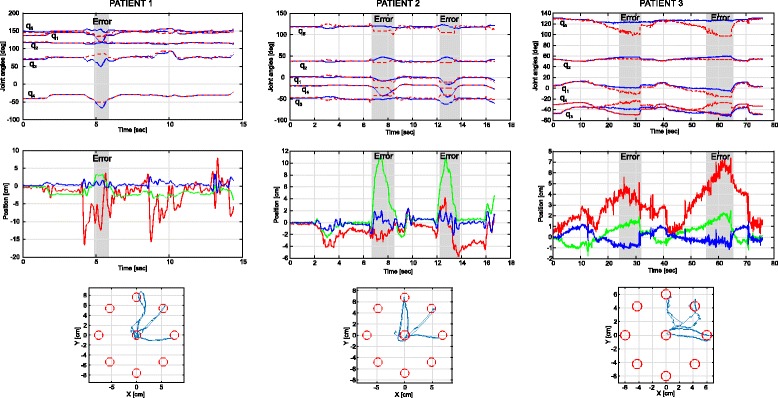
*(Up)* Upper limb joints angles estimated through the proposed algorithm (blue line) and with the previous algorithm (red dotted line). The gray area denote the instability of the previous algorithm, i.e. the time in which the upper limb joints cannot be estimated with the previous algorithm. In these areas the arm joints were set to the last known value estimated through the previous algorithm. *(Middle)* Shoulder displacement performed during the therapy by the patients. The red line is the right(+)/left(-) movement, the green line is the forward(+)/backward(-) movement and the blue line means the up(+)/down(-) movement. *(Down)* Trajectory performed during the therapy with the end effector of the robot (blue line). The red circles are the goals of the roulette exercise

The proposed kinematic reconstruction algorithm was tested in a clinic environment with post-stroke patients during a robot-aided neuro-rehabilitation therapy with the ‘PUPArm’ robot. In addition, the previous algorithm presented by Papaleo et al. was also studied [32]. Figure 7 shows the upper limb joints estimated with the proposed algorithm and with the previous algorithm. Furthermore, the shoulder displacement of the patients and the trajectory followed with the end effector of the robot are also shown. The gray area denote the instability of the previous algorithm, i.e. the time in which the upper limb joints cannot be estimated with the previous algorithm. In these areas the arm joints were set to the last known value estimated through the previous algorithm. The trajectory followed with the end effector of the robot is also shown in the figure together with the eight possible goals of the roulette exercise [43]. In this case the exercise performed was to achieve three goals. It can be observed that the diameter of the roulette is higher in the user one, 15 *cm*, than in the user two and three, 13 *cm* and 12 *cm* respectively, implying higher estimated ROM in joints q_1_ and q_3_ (see Table 5), as it was expected due to the high Fugl-Meyer score (see Table 3).

**Table 5:** ROM, in degrees, estimated through the proposed algorithm during the therapy

ID	Joint q_1_	Joint q_2_	Joint q_3_	Joint q_4_	Joint q_5_
1	37.5	5.46	43.63	35.8	11.78
2	17.99	11.74	18.04	29.29	9.20
3	18.99	9.47	16.13	26.04	11.38

## Discussion

The aim of this study was to develop a robust kinematic reconstruction algorithm of the human upper limb joints being able to perform a real-time joint estimation during a neuro-rehabilitation therapy assisted by robots with only one accelerometer placed onto the upper arm. Furthermore, the previous algorithm, presented in [32], the initial upper limb joints; the upper arm and the forearm length; and the shoulder position were measured by the optoelectronic system, not used in a clinical environment. Therefore, we have defined a protocol to manually measure the upper arm and forearm lengths; we have introduced a mathematical method to estimate the initial upper limb joints; and the presented algorithm, as it is based on the computation of the accelerometer rotation, is always able to estimate the upper limb joints.

The kinematic reconstruction algorithm proposed shows high correlation with respect to the real upper arm. Although the error committed in the estimation of the wrist and shoulder position is low, 2*cm* approximately, it implies an upper limb joints RMSE about 3.5 *degrees* (mean of the seven joints) with high correlation in all joints. It must be noticed that q_5_ and q_6_ joints have low correlation with respect to the others, it may be due to these joints are in the distal part of the arm where the error between the real arm and the estimated arm is maximum and the estimation could differ slightly.

The second experiment was intended to study the behavior of the proposed and previous algorithms in patients during a neuro-rehabilitation therapy assisted by end-effector robots, being able to estimate the shoulder movements using the method proposed in [34] and assuming the joints q_6_ and q_7_ fixed by the robot. Figure 7 shows that the previous algorithm is unstable when shoulder movements appear, areas marked in gray, whilst the proposed not. Although the shoulder is assumed fixed in both methods, it is very difficult to fix the shoulder and avoid little displacements with patients. It must be noticed that, before the error appears, the difference between both algorithms increases and, after the instability, the previous algorithm tends to follow the proposed estimated joints. Therefore, we can say that in the areas when the previous algorithm fails the proposed kinematic reconstruction performs a correct estimation. This error appears due to the method employed in the estimation of the elbow joint location because it is based on the strict constrains of the human upper limb which, a little movement of the shoulder assumed fixed, can lead to the algorithm failure. Furthermore, this error is closely related to the ROM estimation, a very important parameter in these therapies, and could lead a false ROM improvement [17]. Therefore, it is very important the stability of the kinematic reconstruction algorithm during the exercise.

On the other hand, the estimation of the ROM together with the assessments scales proposed and the trajectory performed by the user with the end effector of the robot encompasses an objective and comprehensive assessment of the patient condition during a robot-aided neuro-rehabilitation therapy. Thus, it can be observed that subject 3 performed worse trajectories than the other two subjects as it was expected due to the low score on the Fugl-Meyer scale with high Ashworth score. Furthermore, the patient with higher Fugl-Meyer and less Ashworth scores has the highest estimated ROM.

## Conclusions

The presented kinematic reconstruction algorithm of the human upper limbs has a low error regarding the real arm acquired through an optoelectronic system. This algorithm performs the kinematic reconstruction during the exercise allowing the therapist to correct, in real time, wrong upper limb position. Furthermore, compared to the previous algorithm, it is stable; proposes a protocol to manually measure the upper arm and forearm length; and estimates the initial upper limb joints being able to be used in a clinic environment. In addition, the study of the kinematics in the ‘normal’ model, performed by healthy subjects, during robot-aided rehabilitation tasks could be directly applied in the evaluation of the patients. Finally, the ROM estimation of the upper limb joints together with the assessment scales, as Fugl-Meyer or Ashworth, and the trajectory performed by the patient allows the therapist to have a comprehensive assessment during the therapy.

## Additional files

Additional file 1 Solution of *γ* angle. (PDF 104 kb)

Additional file 2 Estimation of the initial conditions. (PDF 107 kb)

## Abbreviations

DH: Denavit-hartenberg
DoF: Degree of freedom
MSE: Root Mean square error
ROM: Range of motion
SD: Standard deviation
