# Supplementary material for: Human arm joints reconstruction algorithm in rehabilitation therapies assisted by end-effector robotic devices
Source: J Neuroeng Rehabil. 2018 Feb 20;15:10. doi: 10.1186/s12984-018-0348-0 (PMC5819179; doi:10.1186/s12984-018-0348-0)
Supplement: Supplementary file 1 — Solution of γ angle. (PDF 104 kb) [file 12984_2018_348_MOESM1_ESM.pdf]

Additional file 1 — Solution of γ angle

The γ angle is the necessary angle that the matrix ${}^{acc_0}\tilde{R}_{acc}$ must rotate around the gravity vector in order to estimate the elbow joint position. Thus, the plane formed by the X axis and Y axis (plane Π in Fig. 3 of the paper) must include the known wrist (W) and shoulder (S) points. The γ angle can be also computed by translating W to Π around the gravity vector placed in S. Hence, the new wrist point ($\tilde{\hat{W}}$) can be computed as

$$\tilde{\hat{W}} = \left(g \cdot \hat{W}\right) g + \cos\left(\gamma\right) \left(\hat{W} - \left(g \cdot \hat{W}\right) g\right) - \sin\left(\gamma\right) \left(g \times \hat{W}\right),$$

where

$$\hat{W} = \frac{(W - S)}{\| (W - S) \|},$$
$$g = \begin{bmatrix} 0 & 0 & -1 \end{bmatrix}^T.$$

Therefore, the distance between $\tilde{\hat{W}}$ and Π should be zero and is expressed as

$$d\left(\tilde{\hat{W}}, \Pi\right) = \frac{\left|A_\Pi \tilde{\hat{W}}_x + B_\Pi \tilde{\hat{W}}_y + C_\Pi \tilde{\hat{W}}_z + D_\Pi\right|}{\sqrt{{A_\Pi}^2 + {B_\Pi}^2 + {C_\Pi}^2}} = 0; \quad (1)$$

where

$$\begin{bmatrix} A_\Pi \\ B_\Pi \\ C_\Pi \end{bmatrix} = \overline{S\tilde{P}^y_{acc}} \times \overline{\tilde{P}^x_{acc}\tilde{P}^y_{acc}},$$
$$D_\Pi = \begin{bmatrix} A_\Pi & B_\Pi & C_\Pi \end{bmatrix}^T \cdot S;$$

and

$$\tilde{P}^x_{acc} = {}^{acc_0}\tilde{R}_{acc} \begin{bmatrix} 1 & 0 & 0 \end{bmatrix}^T,$$
$$\tilde{P}^y_{acc} = {}^{acc_0}\tilde{R}_{acc} \begin{bmatrix} 0 & 1 & 0 \end{bmatrix}^T,$$
$$\overline{S\tilde{P}^y_{acc}} = \left(\tilde{P}^y_{acc} - S\right),$$
$$\overline{\tilde{P}^x_{acc}\tilde{P}^y_{acc}} = \left(\tilde{P}^y_{acc} - \tilde{P}^x_{acc}\right),$$

From this point, the resolution of γ is computationally expensive and a simplification is needed in order to estimate the upper limb joints in real time.

Thereby, by defining

$$
\begin{aligned}
a &= A_{\Pi} \cdot \tilde{\hat{W}}_x + B_{\Pi} \cdot \tilde{\hat{W}}_y,\\
b &= A_{\Pi} \cdot \tilde{\hat{W}}_y + B_{\Pi} \cdot \tilde{\hat{W}}_x,\\
c &= A_{\Pi} \cdot S_x + B_{\Pi} \cdot S_y + C_{\Pi} \cdot (\tilde{\hat{W}}_z + S_z) + D_{\Pi},
\end{aligned}
$$

and (1) is rewritten as

$$a \cdot cos\,(\gamma) - b \cdot sin\,(\gamma) + c = 0. \tag{2}$$

Then, multiplying and dividing by the norm of vector $\vec{ab}$, (2) yields

$$\sqrt{a^2+b^2}\left[\frac{a}{\sqrt{a^2+b^2}} \cdot cos\,(\gamma) + \frac{-b}{\sqrt{a^2+b^2}} \cdot sin\,(\gamma)\right] + c = 0. \tag{3}$$

On the other hand, as the component of a vector divided by its norm varies between ± 1, the following approximation can be assumed

$$
\begin{aligned}
cos\,(\eta) &= \frac{a}{\sqrt{a^2+b^2}},\\
sin\,(\eta) &= \frac{-b}{\sqrt{a^2+b^2}}.
\end{aligned}
$$

Hence, (3) can be rewritten as

$$
\begin{aligned}
m\,[cos\,(\eta) \cdot cos\,(\gamma) + sin\,(\eta) \cdot sin\,(\gamma)] + c = 0;\\
m \cdot cos\,(\eta - \gamma) + c = 0.
\end{aligned}
$$

being $m = \sqrt{a^2+b^2}$. Thus, the desired γ value remains solved as

$$\gamma = \eta - arcos\left(\frac{-c}{m}\right);$$

where

$$\eta = artg\left(\frac{-b}{a}\right).$$

Finally, two possible γ values are obtained as

$$
\begin{aligned}
\gamma_1 &= artg\left(\frac{-b}{a}\right) - arcos\left(\frac{-c}{\sqrt{a^2+b^2}}\right),\\
\gamma_2 &= artg\left(\frac{-b}{a}\right) - arcos\left(\frac{-c}{-\sqrt{a^2+b^2}}\right) - \pi.
\end{aligned}
$$

These solutions allows us to perform the kinematic reconstruction in real time as the solutions are computed through simple mathematical operations.
