# Supplementary material for: Human arm joints reconstruction algorithm in rehabilitation therapies assisted by end-effector robotic devices
Source: J Neuroeng Rehabil. 2018 Feb 20;15:10. doi: 10.1186/s12984-018-0348-0 (PMC5819179; doi:10.1186/s12984-018-0348-0)
Supplement: Supplementary file 2 — Estimation of the initial conditions. (PDF 107 kb) [file 12984_2018_348_MOESM2_ESM.pdf]

Additional file 2 — Estimation of the initial conditions

The initial upper limb joint are necessary to the kinematic reconstruction algorithm. The following locations with respect to the robot are initially known: the shoulder ${}^{r}T_s$, obtained at the begining of the therapy; the wrist ${}^{r}T_w$, known through the end effector of the robot; and the elbow ${}^{r}T_e$, estimated as explained in the previous section. Thus, the initial joint angles can be estimated using the DH parameters [35] shown in Table 1.

Let's define the homogeneous transform matrix as

$$T = \begin{bmatrix} n_x & n_y & n_z & p_x \\ o_x & o_y & o_z & p_y \\ a_x & a_y & a_z & p_z \\ 0 & 0 & 0 & 1 \end{bmatrix}. \tag{1}$$

Then, shoulder movement can be defined with its homogeneous matrix regarding the joints q_1, q_2 and q_3 as

$$\begin{aligned} {}^{s_0}T_{s_3} &= {}^{s_0}T_{s_1} \cdot {}^{s_1}T_{s_2} \cdot {}^{s_2}T_{s_3}, \\ {}^{s_0}T_{s_3} &= \begin{bmatrix} c_1 s_3 - c_3 s_1 s_2 & -c_2 s_1 & c_1 c_3 + s_1 s_2 s_3 & l_u c_2 s_1 \\ s_1 s_3 + c_1 c_3 s_2 & c_1 c_2 & c_3 s_1 - c_1 s_2 s_3 & -l_u c_1 c_2 \\ -c_2\ c_3 & s_2 & c_2\ s_3 & -l_u s_2 \\ 0 & 0 & 0 & 1 \end{bmatrix}, \end{aligned}$$

with $c_i = \cos(q_i)$ and $s_i = \sin(q_i)$, $i = \{1, 2, 3\}$. This matrix determines the coordinate system s_3 with respect to the system s_0, i.e. the known matrix ${}^{r}T_s$ and, therefore, two possible solutions of the shoulder joints can be obtained, expressed with the nomenclature presented in (1): (i) if $q_2 \in [0 \quad \pi]$

$$\begin{aligned} q_1 &= \operatorname{atan2}(-n_y, o_y), \\ q_2 &= \operatorname{atan2}\left(a_y, \sqrt{n_y^2 + o_y^2}\right), \\ q_3 &= \operatorname{atan2}(a_z, -a_x); \end{aligned}$$

and (ii) if $q_2 \in [-\pi \quad 0]$

$$\begin{aligned} q_1 &= \operatorname{atan2}(n_y, -o_y), \\ q_2 &= \operatorname{atan2}\left(a_y, -\sqrt{n_y^2 + o_y^2}\right), \\ q_3 &= \operatorname{atan2}(-a_z, a_x). \end{aligned}$$

The flexion-extension of the elbow, joint q_4, affects the distance $\overline{SW}$ and, therefore, it can be unequivocally computed through the law of the cosines as

$$q_4 = \arcsin\left(\frac{l_u^2 + l_f^2 - ||W - S||^2}{2 l_u l_f}\right).$$

Since the wrist location is given by the robot end-effector pose, its transformation matrix ${}^{r}T_{w} = {}^{s_0}T_{s_7}$ is known. Thus, the wrist joints can be also estimated following the criterion used to solve the shoulder joints as

$$
\begin{aligned}
{}^{s_4}T_{s_7} &= \left({}^{s_0}T_{s_3} \cdot {}^{s_3}T_{s_4}\right)^{-1} \cdot {}^{s_0}T_{s_7}, \\
{}^{s_4}T_{s_7} &= \begin{bmatrix} c_5c_7 - s_5s_6s_7 & -c_6s_5 & c_5s_7 + c_7s_5s_6 & 0 \\ c_7s_5 + c_5s_6s_7 & c_5c_6 & s_5s_7 - c_5c_7s_6 & 0 \\ -c_6s_7 & s_6 & c_6c_7 & l_f \\ 0 & 0 & 0 & 1 \end{bmatrix},
\end{aligned}
$$

with ${}^{s_3}T_{s_4}$ the homogeneous matrix of the joint q_4. Two possible solutions can be also obtained, the first solution is

$$
\begin{aligned}
q_5 &= -\text{atan2}\left(n_y, o_y\right), \\
q_6 &= \arcsin\left(a_y\right), \\
q_7 &= -\text{atan2}\left(a_x, a_z\right);
\end{aligned}
$$

and, the second solution, due to q_6 is computed through the *arcsin*, is

$$
\begin{aligned}
q_5 &= \pi - \text{atan2}\left(n_y, o_y\right), \\
q_6 &= \pi - \arcsin\left(a_y\right), \\
q_7 &= \pi - \text{atan2}\left(a_x, a_z\right).
\end{aligned}
$$

Thereby, four solutions, two due to the shoulder joints and two due to the wrist joints, can satisfy the kinematic constrains. However, only one solution accomplishes the anatomical features of the human upper limb. This statement is provable because the human arm joints vary in $[-\pi/2 \quad \pi/2]$ and each solution belongs either $[0 \quad \pi]$ range or $[0 \quad -\pi]$ range and, therefore, the initial arm joints remain defined.
